# Endoscopic endonasal double flap technique for reconstruction of large anterior skull base defects: technical note^☆^

**DOI:** 10.1016/j.bjorl.2018.03.008

**Published:** 2018-04-19

**Authors:** Ricardo Landini Lutaif Dolci, Alexandre Bossi Todeschini, Américo Rubens Leite dos Santos, Paulo Roberto Lazarini

**Affiliations:** a Santa Casa de Misericórdia de São Paulo, Faculdade de Ciências Médicas, Departamento de Otorrinolaringologia, São Paulo, SP, Brazil; b Santa Casa de Misericórdia de São Paulo, Faculdade de Ciências Médicas, Departamento de Cirurgia, Disciplina de Neurocirurgia, São Paulo, SP, Brazil

**Keywords:** Cerebrospinal fluid leak, Skull base, Meningioma, Vazamento de líquido cefalorraquidiano, Base do crânio, Meningioma

## Abstract

**Introduction:**

One of the main concerns in endoscopic endonasal approaches to the skull base has been the high incidence and morbidity associated with cerebrospinal fluid leaks. The introduction and routine use of vascularized flaps allowed a marked decrease in this complication followed by a great expansion in the indications and techniques used in endoscopic endonasal approaches, extending to defects from huge tumours and previously inaccessible areas of the skull base.

**Objective:**

Describe the technique of performing endoscopic double flap multi-layered reconstruction of the anterior skull base without craniotomy.

**Methods:**

Step by step description of the endoscopic double flap technique (nasoseptal and pericranial vascularized flaps and fascia lata free graft) as used and illustrated in two patients with an olfactory groove meningioma who underwent an endoscopic approach.

**Results:**

Both patients achieved a gross total resection: subsequent reconstruction of the anterior skull base was performed with the nasoseptal and pericranial flaps onlay and a fascia lata free graft inlay. Both patients showed an excellent recovery, no signs of cerebrospinal fluid leak, meningitis, flap necrosis, chronic meningeal or sinonasal inflammation or cerebral herniation having developed.

**Conclusion:**

This endoscopic double flap technique we have described is a viable, versatile and safe option for anterior skull base reconstructions, decreasing the incidence of complications in endoscopic endonasal approaches.

## Resumo

**Introdução:**

Uma das principais preocupações em abordagens endoscópicas endonasais da base do crânio tem sido a alta incidência e morbidade associada a fístulas liquóricas. A introdução e o uso rotineiro de retalhos vascularizados permitiram uma acentuada redução dessa complicação, seguida por uma grande expansão nas indicações e técnicas utilizadas nas abordagens endoscópicas endonasais, incluindo grandes tumores e áreas anteriormente inacessíveis da base do crânio.

**Objetivo:**

Descrever a técnica cirúrgica realizando uma reconstrução endoscópica multicamadas da base anterior do crânio com duplo retalho, sem craniotomia.

**Método:**

Descrição passo a passo da técnica endoscópica com duplo retalho (retalhos vascularizados nasoseptal e pericraniano e enxerto livre de fascia lata), utilizados e ilustrados em dois pacientes com meningioma do sulco olfatório submetidos à cirurgia por via endoscópica endonasal.

**Resultados:**

Em ambos os pacientes procedeu-se ressecção total macroscópica seguido de reconstrução da base anterior do crânio com os retalhos nasoseptal e pericraniano *onlay* e enxerto livre de fáscia lata *inlay*. Os pacientes apresentaram uma excelente recuperação, sem sinais de fístula liquórica, meningite, necrose do retalho, inflamação meníngea crônica ou sinonasal ou hérnia cerebral.

**Conclusão:**

A técnica endoscópica de duplo retalho, como descrita, trata-se de uma opção viável, versátil e segura para as reconstruções da base anterior do crânio, diminuindo a incidência de complicações em abordagens cirúrgicas endoscópicas endonasais.

## Introduction

Different types of vascularized flaps, nasal and extranasal, have been described for different objectives, such as facial reconstruction, orbital elevation, septal perforation corrections and, mainly, for the closure of cerebrospinal fluid (CSF) leaks during and postoperatively of the endoscopic endonasal skull base surgery, allowing for fewer technique-related complications and an impressive growth of this technique.^1^, ^2^, ^3^, ^4^, ^5^, ^6^ The choice of vascularized flap to be used is related to the size of the expected skull base defect, previous surgeries, tumour type (benign or malignant), location and tumour extension to possible donor areas.^4^

The more widely used nasal flaps are the (1) nasoseptal, (2) inferior turbinate, (3) middle turbinate and (4) lateral wall. The extra-nasal flaps are the (1) pericranial, (2) temporoparietal fascia and (3) palatal.^2^, ^4^, ^7^, ^8^, ^9^, ^10^, ^11^

The nasoseptal flap can be considered a milestone in the development and growth of the endoscopic endonasal surgery of the skull base and it has become the workhorse to close the CSF leaks inherent to this technique. It is easy to obtain and versatile, reaching from the clival region to the anterior fossa.^1^, ^4^

However, there are some tumours and approaches that create an extensive skull base defect that is not possible to be closed using only the nasoseptal flap, requiring either a free graft or a second vascularized flap.

The vascularized flap with best results for anterior skull base defects is the extra nasal pericranial flap that usually requires a craniotomy. This study aims to show a novel technique for the use of the pericranial flap without a craniotomy, alongside the nasoseptal flap and a free fascia lata graft, for the correction of extensive anterior skull base defects, yielding a lower complication rate.^10^, ^12^, ^13^

## Methods

We retrospectively reviewed the chart information of two patients with an olfactory groove meningioma diagnosis who underwent endoscopic endonasal approach for tumour resection and skull base reconstruction using the technique we will outline, using double vascularized flaps (the nasoseptal and the pericranial) and a free fascia lata graft. This study was reviewed and approved by our institution's Ethics Committee (CAAE 63767417.0.0000.5479).

### Patient 1

**Figure 1:**
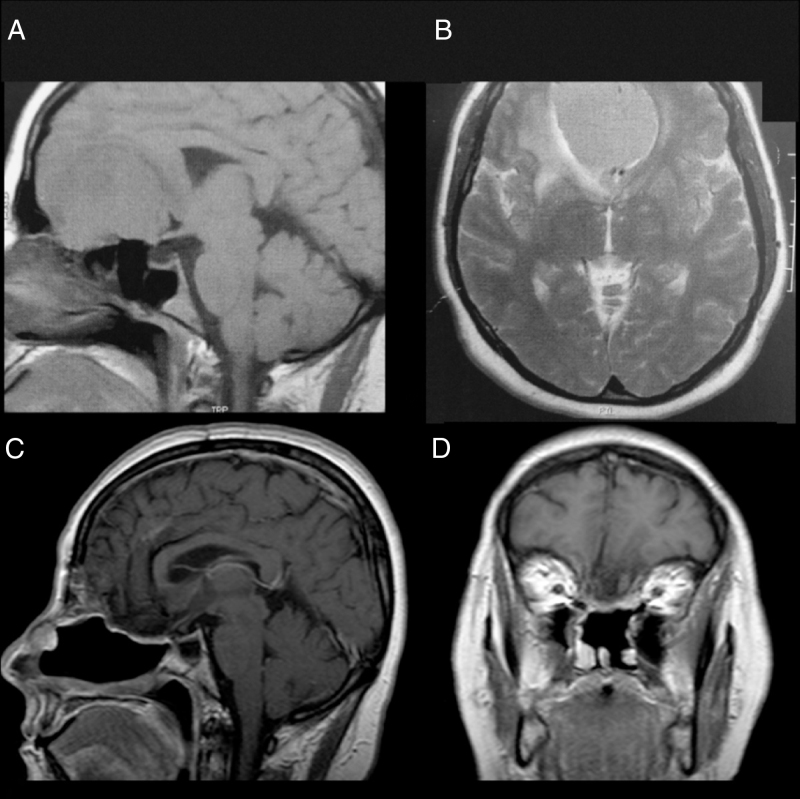
Patient 1, female, 49 years old. (A, B) Preoperative MRI without a contrast showing a large mass in the anterior fossa with mass effect. The anterior cerebral arteries are displaced posteriorly and there is invasion of the anterior and posterior ethmoid. (C, D) Postoperative 4 years MRI with contrast, showing gross total resection and no signs of tumour recurrence or brain herniation. Both flaps can be seen with gadolinium enhancement and preservation of the pedicles and viability of the endoscopic double flap.

Female, 49 years old, complaining of diminished olfaction and cognitive changes (periods of disorientation and confusion and memory impairment). Magnetic resonance imaging (MRI) showed a large intracranial mass, with dural attachment and tail on top of the cribriform plate, occupying most of the anterior skull base with significant mass effect and oedema in both frontal lobes (Fig. 1).

### Patient 2

**Figure 2:**
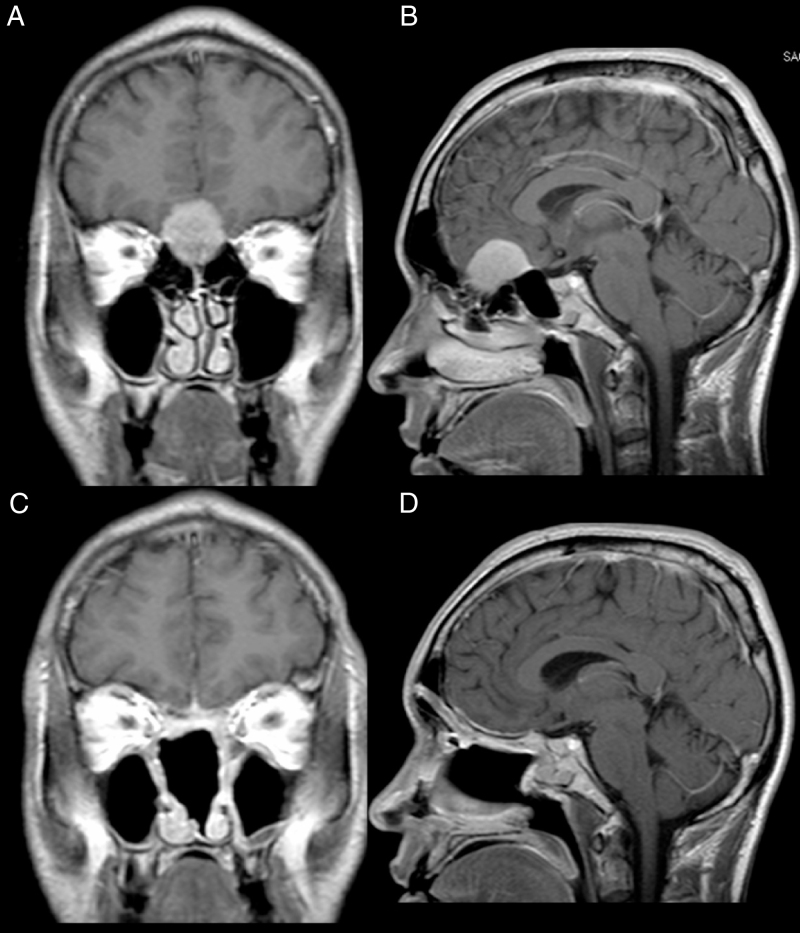
Patient 2, male, 39 years old. (A, B) Preoperative MRI with contrast showing an intracranial mass with dural attachment and invasion of the anterior and posterior ethmoid. (C, D) Postoperative 1 year MRI with contrast, showing gross total resection and no signs of tumour recurrence or brain herniation. Both flaps can be seen with gadolinium enhancement and preservation of the pedicles and viability of the endoscopic double flap.

Male, 39 years old, complaining of mild to moderate frequent headaches and diminished olfaction. MRI showed an intracranial mass with dural attachment on top of the cribriform plate with bone invasion of the plate and the ethmoid (Fig. 2).

## Operative technique

### The nasoseptal flap

In both cases the nasoseptal flap was harvested from the right side, employing the standard upper incision is with care to preserve the superior portion (about 15 mm) of the septal mucosa, from its posterior margin to the middle turbinate, in order to preserve the delicate olfactory nerve filaments present in this area and, therefore, the patient's olfaction.^1^, ^3^ However, in both cases a transcribriform approach was planned, which leads to postoperative anosmia. The upper incision was made higher, without care for the olfactory mucosa, in order to maximize the available area of the flap. The inferior incision was also lowered to the floor of the nasal cavity with the same objective.

In addition, was removed the vomer and the perpendicular plate of the ethmoid. To protect the exposed septum cartilage and to reduce postoperative crusting, a reverse flap using the contralateral mucosa was fashioned.

### Tumour removal

The anterior and posterior ethmoidal arteries were ligated and cut bilaterally, interrupting part of the arterial supply to the tumour, reducing intraoperative bleeding. An endonasal craniectomy was performed in the roof of the nasal cavities, including the removal of the cribriform plate, for a greater reach and tumour exposure. The limits of the opening are the posterior wall of the frontal sinus (anterior), the planum sphenoidale (posterior) and the transition between the cribriform plate and the fovea ethmoidalis (lateral). Next, the dura mater was opened and the tumour debulked, allowing easier manipulation of the remaining tumour so it could be carefully separated from the normal brain, using a bimanual dissection technique by the surgeon with the assistant responsible for handling the endoscope and, when necessary, irrigation or suction of the cavity (4-hand endonasal surgery). This technique allows a gross total removal of the tumour and its capsule, while respecting and preserving the plane of dissection provided by the arachnoid membrane, that protects the delicate perforating vasa and nervous tissue. In both cases, a gross total resection was achieved although the preservation of the olfactory nerves was not possible.

### The pericranial flap

A bicoronal incision was performed, approximately 1.5 cm anterior to each external acoustic meatus and crossing the scalp posteriorly to the coronal sutures. After this initial incision, the skin flap is elevated anteriorly up to about 10 mm above the orbital rim, through the loose areolar layer, a typically avascular layer reducing bleeding and blood loss. The pericranium and temporal muscles were kept attached to the skull at this time.

Care should be taken to avoid separating the skin flap and the pericranium beyond 10 mm above the orbital rim due to the risk of injury the neurovascular supply of the skin and pericranium. These are supplied by the superficial branches of the supra-orbital and supratrochlear nerves and the supra-orbital and supratrochlear arteries and its branches (superficial branches to the skin and deep branches to the flap).^14^ In order to avoid muscle atrophy, reduce postoperative pain and a produce a better aesthetic outcome we avoid elevating or cutting the temporalis muscles.

Once the posterior extent of the pericranial flap is satisfactory, lateral incisions are made immediately above the superficial temporal lines and connected posteriorly. The pericranial flap is then elevated anteriorly from the skull all the way to the orbital rims, leaving the last 10 mm still attached to the skin (both skin an pericranium are elevated from the skull together beyond this point).

**Figure 3:**
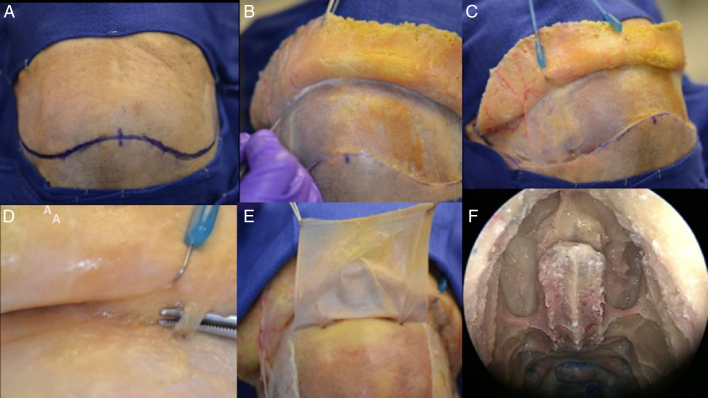
Step by step of a dissection showing how to raising the pericranial flap, the pictures was done in a cadaver. (A) Bicoronal incision was performed, approximately 1.5 cm anterior to each external acoustic meatus and crossing the scalp posteriorly to the coronal sutures. (B) After this initial incision, the skin flap is elevated anteriorly up to about 10 mm above the orbital rim, through the loose areolar layer, a typically avascular layer reducing bleeding and blood loss. The pericranium and temporal muscles were kept adhered to the skull at this time. (C) In a lateral view, it is feasible to visualize the left superficial temporal artery and the fascia exposed. (D, E) This flap is supplied by the supra-orbital and supratrochlear arteries and its branches (superficial branches to the skin and deep branches to the flap), in the picture is emphasized the supraorbital nerve. The pericranial flap is then elevated anteriorly from the skull all the way to the orbital rims, leaving the last 10 mm adhered to the skin (both skin an pericranium are elevated from the skull together beyond this point). (F) In the nose, it is necessary to prepare the way to receive the flap and avoid any infection or Mucocele posteriorly, so it is mandatory to perform a Draf III (modified endoscopic Lothrop) allowing the passage of the pericranial flap posteriorly to the skull base to the nasal cavity. The Draf III is the communication of the right and left frontal sinus and removing any internals septa.

**Figure 4:**
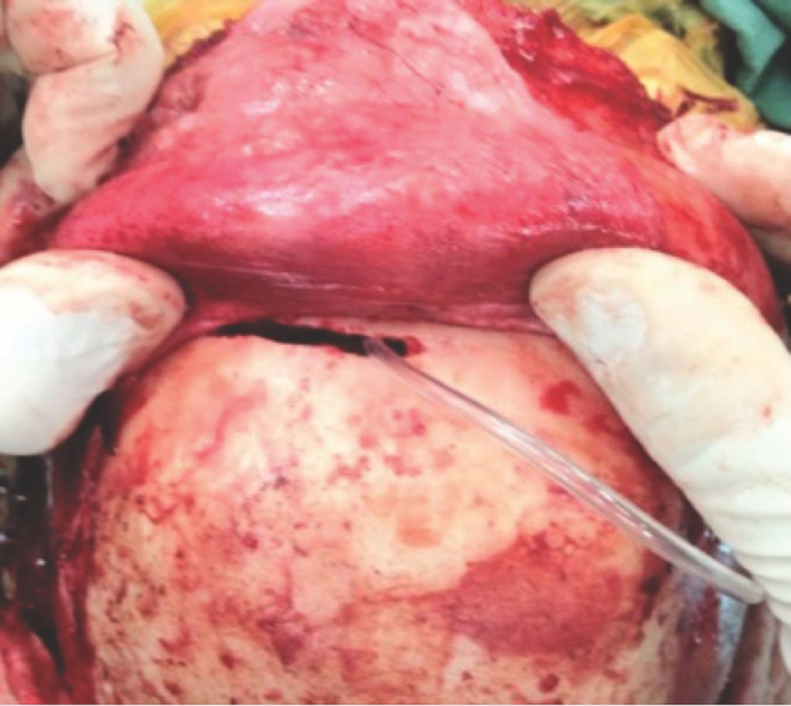
Intraoperative picture showing the ‘window’ created in the anterior wall of the frontal sinus for passage of the pericranial flap.

Being an extranasal flap it is necessary to make an opening in the glabella, in the anterior wall of the frontal sinus, about 20 mm × 5 mm, so the flap can reach the nasal cavity through the frontal sinus. A Draf III (modified endoscopic Lothrop) must be done to communicate the frontal sinus and remove any internal septa, allowing the passage of the pericranial flap posteriorly to the skull base and nasal cavity. Doing this technique, it is feasible to avoid any infection and mucocele, keeping the frontal sinus widely open to remove the mucosa inside (Figure 3, Figure 4).

If necessary, lateral incisions at the anterior portion of the flap can be performed to ease its passage through the narrow window opened in the anterior wall of the frontal sinus. However, these must be done very carefully to avoid injury to the vascular pedicle of the flap.

### Skull base reconstruction

**Figure 5:**
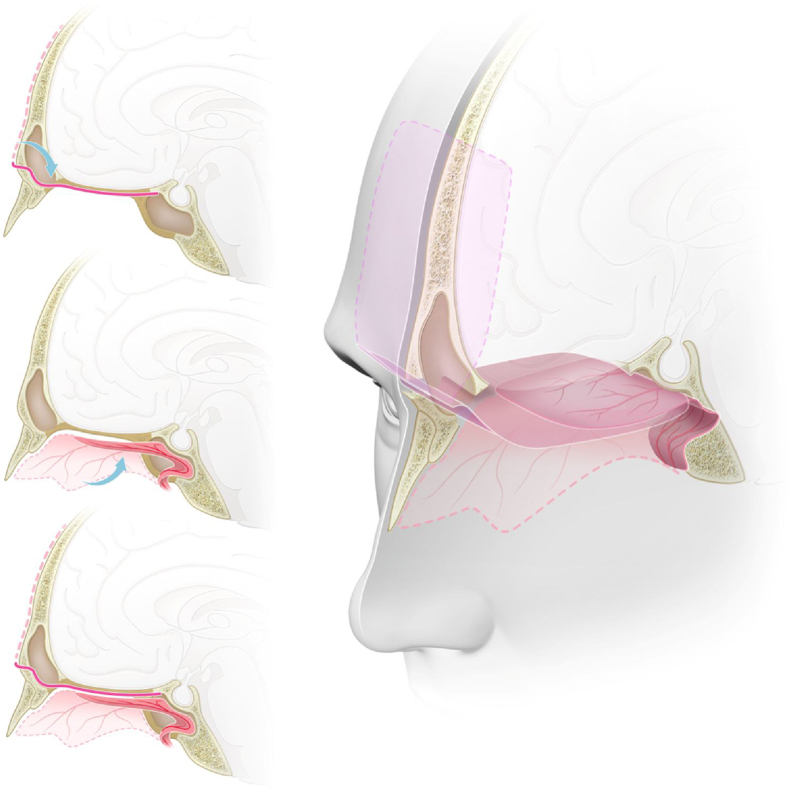
Graphic representation of the double flap technique with the pericranial and nasoseptal flaps for reconstruction of the anterior skull base. (A) Sagital view. The pericranial flap, after being harvested with a bicoronal incision, is introduced to the sinonasal cavity through a ‘window’ in the anterior wall of the frontal sinus reaching the planum sphenoidale and the sella. (B) Sagital view. Nasoseptal flap harvested from the medial wall of the nasal cavity, preserving its vascular pedicle, and placed over the anterior skull base defect. (C) Sagital view. Both pericranial and nasoseptal flaps overlaying and reinforcing each other's weak points for the reconstruction of the anterior skull base, reducing the risk of CSF leak or brain herniation. (D) Tridimensional view with a sagittal midline section. Skull base defect from the posterior wall of the frontal sinus to the planum sphenoidale, reconstructed using the endoscopic double flap technique with the pericranial and nasoseptal flaps reinforcing each other, improving the mechanical support for intracranial structures and a more effective barrier to CSF.

Initially, a free fascia lata graft was harvested from the right thigh and placed inlay at the skull base opening, covering all the defect. Then, the pericranial flap, coming from the frontal sinus, was placed onlay over the anterior fossa, from the posterior wall of the frontal sinus heading posteriorly, and the nasoseptal flap was placed over the sphenoid moving anteriorly over the defect, Lastly, the pericranial flap with fibrin sealant was applied. That way, a multi-layered reconstruction was achieved with a double-vascularized flap over the defect, with each flap being reinforced by the other at its free margin (Fig. 5). We had not used the lumbar drain in those cases.

## Results

Both patients underwent successful endoscopic endonasal transcribriform approaches with gross total resection and a double flap anterior skull base reconstruction using a free fascia lata graft inlay, the pericranial flap (harvested with a bicoronal incision and inserted to the nasal cavity through a window in the anterior wall of the frontal sinus) and the nasoseptal flap, both covering the defect, forming a 3-layered reconstruction with a double vascularized flap.

Neither patient had postoperative CSF leak or meningitis. During follow up (4 years for patient 1 and 1 year for patient 2) synechiae, epistaxis or mucocele did not develop and both flaps showed good viability without signs of necrosis.

**Figure 6:**
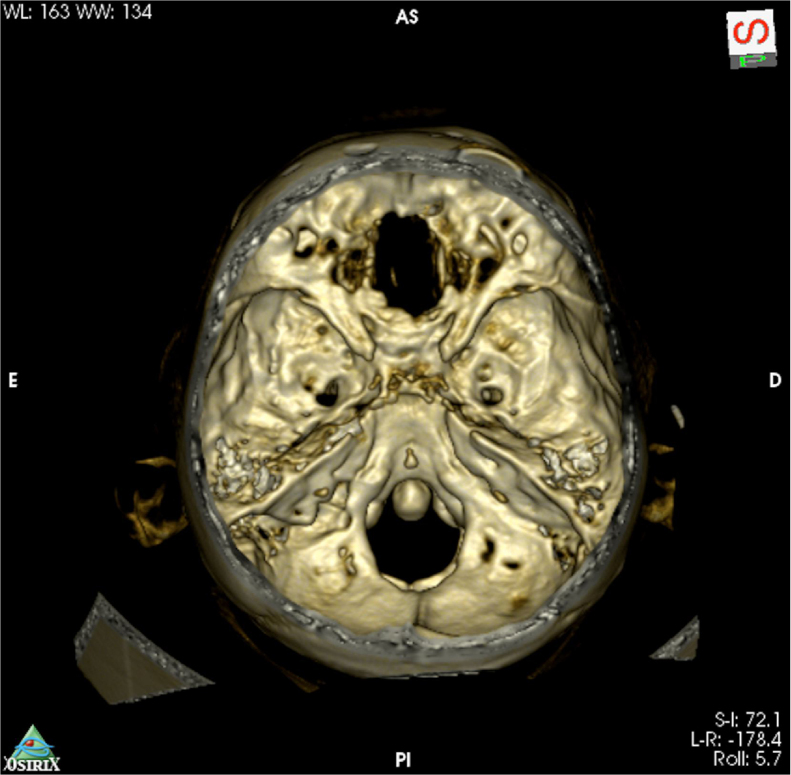
Tridimensional reconstruction of postoperative high definition head CT with a large bone defect from the planum sphenoidale to the crista galli.

Both patients had anosmia after the procedure as expected for a transcribriforme approach, but were free of recurrence and without any signs of brain herniation through the bone defect on magnetic resonance imaging (Figure 1, Figure 2, Figure 6).

## Discussion

The endoscopic endonasal approach for olfactory grooves meningiomas has many advantages over traditional craniotomies. Helpful is early devascularisation of the tumour with the bilateral ligation of the anterior and posterior ethmoid arteries, which are responsible for the arterial irrigation of the tumour.^15^, ^16^

A lower recurrence rate occurred thanks to a true Simpson Grade 1 resection,^17^ with removal of the affected bone and dura mater. These have to be removed in order to reach the tumour via the endoscopic approach and when preserved during traditional craniotomies they are frequent tumour recurrence locations.^18^, ^19^ As shown by Nanda et al., in 458 intracranial meningiomas of different locations during a 20 years follow up, when a Simpson Grade 1 resection was possible the recurrence rate was 5% and those with a Simpson Grade 2 resection, recurrence was 22%.^20^

There is no need for brain retraction, once the craniectomy and dural opening are performed directly adjacent to the tumour, without normal brain between the craniotomy and the tumour. De Almeida et al. in 2015, showed a study that when compared to the traditional bifrontal craniotomy, the endoscopic endonasal approach had significantly less oedema in the adjacent brain, showing as hyperintensity on the FLAIR sequence of the postoperative MRI. Such a difference was attributed to the brain retraction necessary in the traditional bifrontal craniotomy approach and it is related to post-operative ischaemia and haemorrhages and long term cognitive changes.^21^, ^22^

The main concern with the endonasal approach is the high rate of postoperative CSF leak. Before the use of vascularized flaps these rates were between 30% and 40%.^3^, ^18^, ^23^ Van Gompel et al. published a meta-analysis showing a CSF leak for this approach of 26%^24^; de Almeida and colleagues showed an incidence of 30%^21^; Prevedello et al., in 2015, 27.8%^22^ and the Pittsburgh Skull Base Team, 30%.^18^ Most of these studies included or were based on data previous to the routine use of vascularized flaps. More recent studies, based on data with vascularized flaps, show CSF leak rates at 16.1–20%. As the surgical team gains more experience and familiarity with these procedures, the CSF leak rates tend to greatly decrease.^18^, ^19^, ^25^, ^26^

With this in mind, in anterior skull base surgeries with an extensive defect the use of a vascularized flap is paramount to correct and avoid CSF leaks and postoperative meningitis. The nasoseptal flap is not always available with sufficient size to cover the defect. In this study, we present two patients who underwent a successful endoscopic endonasal approach with skull base reconstruction using the double vascularized flap technique. Such technique had already been previously suggested,^12^, ^13^, ^27^, ^28^ however always when using a craniotomy for the pericranial flap. We here describe our technique without need for a craniotomy, using only a small ‘window’ (20 mm × 5 mm) in the anterior wall of the frontal sinus (Fig. 3) with a Draf III^16^, ^29^ to reach the nasal cavity and the anterior fossa defect.

The double flap technique is a complementary reconstruction for extensive defects, in which each flap strengthens the other's weak points as well as the most likely places for CSF leaks. The nasoseptal flap has the anterior part of the defect, close to the frontal sinus its weakest point, while the pericranial flap is greatly reinforced in this area. Complementary, the pericranial flap has its weakest area in the posterior part of the defect where the nasoseptal flap is reinforced. Therefore, their combined use leaves no inherent weak points and further reduces the incidence of CSF leaks.

We also chose to use an inlay fascia lata graft for greater rigidity and support to avoid a possible frontal lobe herniation. Even though it is a rare event, herniation is documented in the literature^30^ and its main risk factors are increased intracranial pressure, usually related to an elevated BMI with or without obstructive sleep apnea (OSA).^31^ The use of rigid autologous (e.g. bone or cartilage) or heterologous (e.g. titanium mesh) are controversial and should be regarded as a last resort due to its higher rates of infection and extrusion of such materials leading to even greater complications.^30^

Another benefit of the double flap is faster healing given the increased blood supply to the area, allowing complementary therapies (e.g. radiotherapy) to be started sooner. The patients here presented had no indication for it, but in malignant tumours where time between surgery and radiotherapy may be an important prognostic factor it is another advantage of this technique.^12^

Harvesting the pericranial flap requires a bicoronal incision. However, since there is no muscle or bone flap removed or even manipulated, healing tends to be without complications, with a very good aesthetic result. The bone window opened in the frontal sinus is covered with the skin and filled with the flap, leaving no impression or external mark. There is description of an endoscopic harvesting of the pericranial flap using three small incisions (at the glabella and in the scalp on either sides)^10^ however it is a time-consuming technique with little advantage to the aesthetic final result.

There are some important restrictions to choosing this approach, such as an unfamiliarity of many neurosurgeons with it, lateral extension of the tumour and a preserved olfaction.

This neurosurgeon unfamiliarity can be overcome by training in experimental conditions in a lab, training courses, accompanying a more experienced neurosurgeon in this kind of procedure and starting with smaller and simpler endonasal procedures. Even then, long learning curve can be long.

The lateral extension can be helped by the use of angled scopes and adequate instruments. If even under optimal conditions a gross total resection cannot be achieved with an endonasal approach, a second stage approach can be used this being a traditional craniotomy which will find the brain more relaxed, improved surgical corridor, smaller tumour and with no need or little need for retraction.

A preserved olfaction does not preclude the endonasal transcribriform approach, but the patient should be made aware that the chances of preserving olfaction are minimal and the impact it may have (e.g. for sommeliers, food critics and related professions). It is important to remember that the traditional technique also has a risk for postoperative anosmia, although smaller than the endonasal technique.^18^, ^19^, ^21^

## Conclusion

The endoscopic endonasal approaches to the anterior skull base have many advantages over traditional craniotomies; however the elevated rates of CSF leaks and associated complications were always a major concern. The use of vascularized flaps has greatly reduced these complications and we present this technique, that we consider it to be a safe and effective option, with the endoscopic double flap (pericranial and nasoseptal), reinforced with a fascia lata free graft inlay, as an alternative to further reduce the complications of this approach.

## Conflicts of interest

The authors declare no conflicts of interest.
